# Developing a framework for monitoring the stages towards achieving effective coverage and equity for maternal, newborn, child health and nutrition interventions

**DOI:** 10.1136/bmjgh-2024-016494

**Published:** 2025-04-09

**Authors:** Patricia S Coffey, Megan E Parker, Katharine D Shelley, Elan Ebeling, Lydia Nguti, Sophia Knudson, Nesibu Agonafir, Sali Ahmed, Savitha Subramanian, Kimberly Mansen, Sadaf Khan, Cyril Engmann, Jessica C Shearer

**Affiliations:** 1 PATH, Seattle, Washington, USA; 2 University of Washington, Seattle, Washington, USA; 3 Bill & Melinda Gates Foundation, Seattle, Washington, USA

**Keywords:** Health systems, Maternal health, Child health, Health policy, Global Health

## Abstract

Reaching the Sustainable Development Goal 2030 global mortality and morbidity targets will require increased access to essential health services. Scaling high-impact health interventions within the public sector is complex; delineation of the pathway to scale for each intervention within each distinct geography is important for prioritising actions to advance interventions towards effective coverage. Following a review of 38 theoretical frameworks describing pathways for scaling health system interventions, we developed, tested and refined a new schema—the Stages of Achieving Effective Coverage and Equity Framework—for use to describe the status of policy adoption, implementation and coverage of key maternal, newborn, child health and nutrition (MNCHN) interventions. We propose a framework with six domains (global, national, systems, implementation, availability and coverage) covering 26 critical milestones with identified corresponding, intervention-specific indicators. Our framework was validated by the alignment of over 83 000 data points sourced from document review, interviews and global or country surveys. Visualisations are presented to highlight how the framework is operationalised to assess scale-up progress. We outline our process of conceptualising and developing a new framework and articulate its use case for action-oriented monitoring of progress towards effective coverage through applied examples in key geographies. Our framework offers an easy-to-follow implementation pathway and a set of common policy and implementation indicators to monitor scale-up towards effective coverage that uses existing secondary data sources where available. Achieving prioritised maternal, newborn and child health targets requires scalable implementation strategies for lifesaving interventions, alongside monitoring progress towards achieving scale.

**SUMMARY BOX**

- The proposed framework was developed to address the need for an easy-to-follow implementation framework and a set of common policy and implementation indicators for monitoring scale-up towards effective coverage, using existing secondary data sources where available.
- The framework is organised around six key domains—global guidelines and market availability, national policy adoption, system integration and readiness, implementation and service delivery, product/service availability and effective coverage—and includes 26 milestones (and intervention-specific corresponding indicators) that capture components typically necessary to achieve (and measure) effective coverage.
- The framework can also be used to customise (eg, at the subnational level) a planning and monitoring approach tailored to where more focus is needed either through pinpointing where progress has stalled, and/or where data gaps indicate more resources are required to measure key milestones along the pathway to scale—both of which can help inform intervention-specific roadmaps, costed plans and further action planning.

## Introduction

 The Sustainable Development Goals (SDGs) are centrally framed around reproductive, maternal, newborn and child health and equity^1^ and provide a clear roadmap to guide progress towards attaining critical global mortality and morbidity targets by 2030. Yet almost half of the world’s population does not have access to essential health services,^2^ decreases in healthcare utilisation during the COVID-19 pandemic have threatened progress in reducing maternal and child mortality,^3^ funding for primary healthcare, including maternal and child health, remains inadequate, inefficient, and fragmented,^4^ and progress towards meeting SDG targets has stalled despite the fact that most maternal and newborn deaths are preventable or treatable.^5^ To get back on track, scaling high-impact interventions to improve maternal, newborn and child survival is critical and will require a thorough examination of the barriers and enablers of scale and investment in tailored implementation strategies to improve the availability and uptake of life-saving interventions.^6 7^

Identification of bottlenecks across the process of introducing and scaling up key health interventions helps to target limited resources and improve availability and coverage. Strengthening supply chains to ensure access to essential medicines and the use of new and underused evidence-based interventions have been identified as key transformative innovations in the SDG era to improve reach and scale.^8 9^ Despite innovations in logistics and practices around essential, evidence-based interventions, significant gaps remain in availability and coverage.^10^

We set out to describe our process of developing a user-friendly framework to support countries in advancing scale-up, by detailing the stages/pathways from global guidelines and market availability to systems readiness and implementation factors necessary for reaching effective coverage, moving beyond access to care to consider key components necessary for achieving quality of care. We contextualise this framework within the health systems literature and a variety of conceptual models and articulate its use case for action-oriented monitoring of progress towards effective coverage.

## The framework: stages of achieving effective coverage and equity

Scaling high-impact health commodities and interventions within the public sector is complex, and defining the pathway to scale is challenging due to heterogeneity, the need to capture different contexts and interventions in a one-size-fits-all model, and the absence of intervention-specific data within country geographies. We developed a standardised and action-oriented framework to aid in comprehensive understanding and visualisation of pathways to scale, which can serve to compare across countries, across interventions and over time. As the initial activity within the Asset Tracker initiative, funded by the Bill & Melinda Gates Foundation, the framework was developed rapidly and efficiently populated with existing secondary data sources to compile a dataset for comparative analyses of progress towards scale.^11^ Our aim was to use a pragmatic framework to identify key domains and their intrinsic milestone activities to pinpoint more effective targeting of limited public health funding towards achieving intervention scale-up. The framework and underlying dataset were subsequently converted into an interactive, web-based dashboard for data visualisation and exploration.^11^

Many holistic multilevel frameworks have been developed and adapted to capture specific barriers and enablers to implementation and scale at all levels of the health system. We conducted a rapid scan of existing theoretical frameworks and models that aim to describe a pathway for scaling up high-impact health interventions to achieve effective and equitable coverage. Among the 38 frameworks sourced and reviewed, five were critical in informing the development of our new framework: the International Development Innovation Alliance model for scaling up innovation,^12^ the Primary Health Care Performance Initiative conceptual framework,^13^ the WHO ExpandNet framework,^14^ the Consolidated Framework for Implementation Research^15^ and the WHO health systems building blocks framework^16^ (see online supplemental file for the 38 frameworks). The five frameworks were selected through expert consensus among the project team based on frameworks commonly applied in our area of public health practice to promote the scale-up of interventions. Based on our framework review and knowledge of implementation strategies for delivering maternal, newborn, child health and nutrition (MNCHN) interventions, we developed a six-stage process framework, characterised by the following domains: (1) global guidelines and market availability, (2) national policy adoption, (3) system integration and readiness, (4) implementation and service delivery, (5) availability and (6) coverage.

We integrated the relatively new measure of effective coverage into our framework as a natural endpoint in the scale-up process. While intervention coverage is assumed to be a ‘crude’ or unadjusted measure of receipt of service, effective coverage moves beyond this in considering aspects of quality that contribute to the actual health benefit of the interventions.^17^ Building from prior research, we used the standardised health-service coverage cascades developed by WHO and UNICEF applied at the population level to visualise scale.^18 19^ This approach allows us to view scale as not only a process of achieving coverage but also of advancing equity and quality.

**Figure 1:**
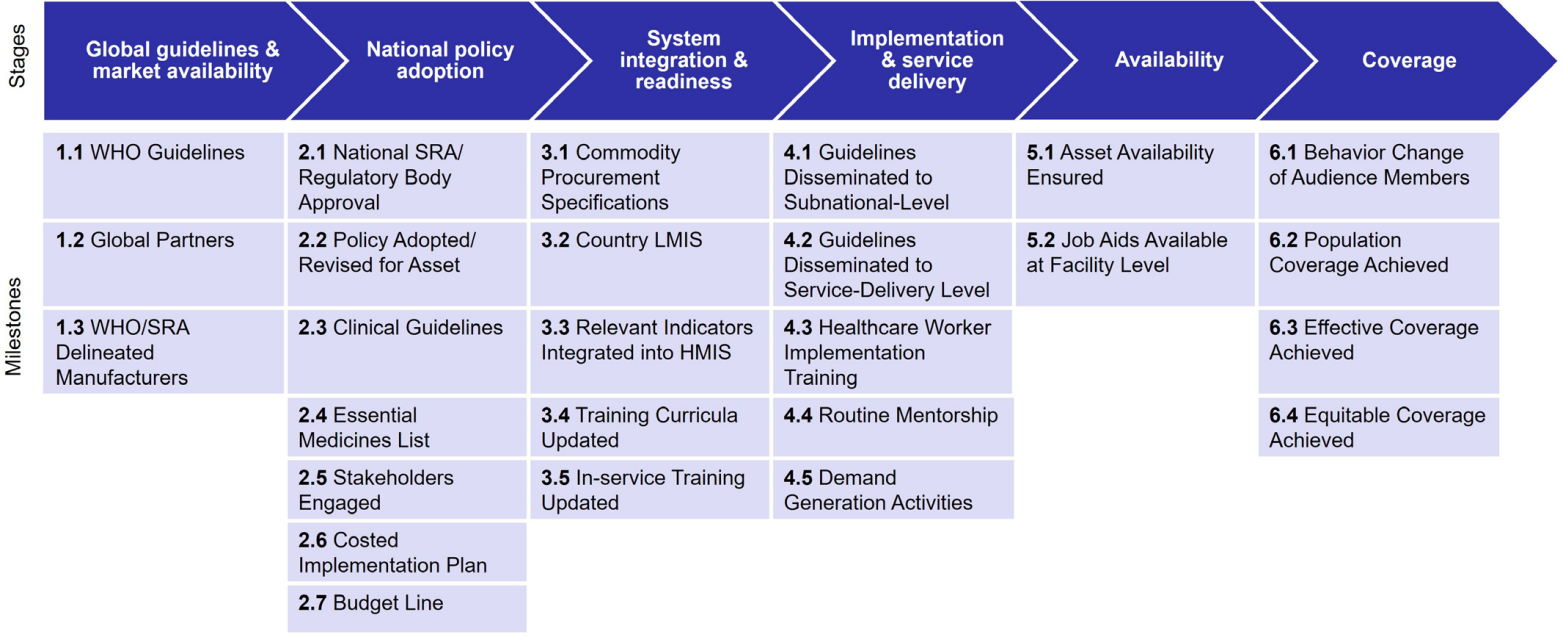
Stages of achieving effective coverage and equity framework. HMIS, health management information system; LMIS, Logistics Management Information Systems; WHO, World Health Organization; SRA, Stringent Regulatory Authority.

This framework illustrates ‘achieving scale’ as a process, showcasing equitable and effective coverage as the final endpoint. We identified 26 key milestones that interventions typically achieve to reach effective coverage (figure 1)—the early-stage milestones serve as explanatory factors contributing to reaching effective and equitable coverage. For each milestone, we identified existing indicators and data sources (documents, surveys, routine data) or collected qualitative responses via global-level and national-level key informant interviews. The framework domains, detailed milestones and intervention-specific indicators were initially vetted during consultations with the donor. They were subsequently validated with stakeholders in Kenya and Ethiopia engaged for input on the design of the interactive dashboards. Although depicted linearly in the framework, we acknowledge that implementation does not always unfold in a linear process across these milestones. Additionally, assessing the key enablers and barriers to achieving milestones is critical to understanding progression (see below for more on operationalising the framework).

## Operationalising the framework

In developing and operationalising the framework, our objectives were to (1) rapidly describe the status of implementation and coverage of selected high-impact maternal, newborn, and child health and nutrition interventions in countries included in the ‘Countdown to 2030 Initiative’,^20 21^ (2) explain barriers and enablers to implementation and coverage in select focus countries (Burkina Faso, Ethiopia, India, Kenya, Malawi, Nigeria, Tanzania and Pakistan—selected by the donor based on their prioritised geographies) and (3) identify strategies to accelerate achievement of policy adoption, implementation and coverage.

To populate the framework milestones with data, we conducted document scans and literature reviews on implementation barriers and enablers of reaching effective coverage (excluding studies on effectiveness), key informant interviews with a purposive sampling of global and country-based experts, and reviewed global surveys and databases to collect and visualise qualitative and quantitative data related to each intervention using various techniques. Literature reviews summarised barriers and enabling strategies from the grey literature and peer-reviewed articles, published journal articles sourced from PubMed, issue briefs and reports from non-governmental organizations (NGOs), country governments and stakeholders (national policies, including clinical guidelines, etc), and global guidelines from normative bodies. Global key informant interviews were conducted to summarise barriers, enabling strategies, future opportunities and existing data from experts working at global normative bodies, international NGOs, professional societies and universities. National (country) key informant interviews summarised local barriers and effective strategies from ministries of health, implementing partners and academic researchers. Last, global surveys and databases were reviewed to obtain indicators including: Demographic and Health Survey (DHS); Service Provision Assessment (SPA); WHO Service Availability and Readiness Assessment (SARA); Every Newborn Action Plan (ENAP), Global Health Observatory; WHO Sexual, Reproductive, Maternal, Newborn, Child, and Adolescent Health (SRMNCAH) Policy Survey; WHO/UNICEF Global Breastfeeding Collective Scorecard; USAID/MOMENTUM Global Survey on National Programs for the Prevention and Management of Postpartum Hemorrhage and Hypertensive Disorders of Pregnancy;^22^ and UNICEF Nutridash. Where accessible, national survey data from select focus countries were included in the database, for example, including the Harmonized Health Facility Assessment (Kenya, Burkina Faso, Ethiopia) and Emergency Obstetric and Newborn Care Survey (Ethiopia).

**Figure 2:**
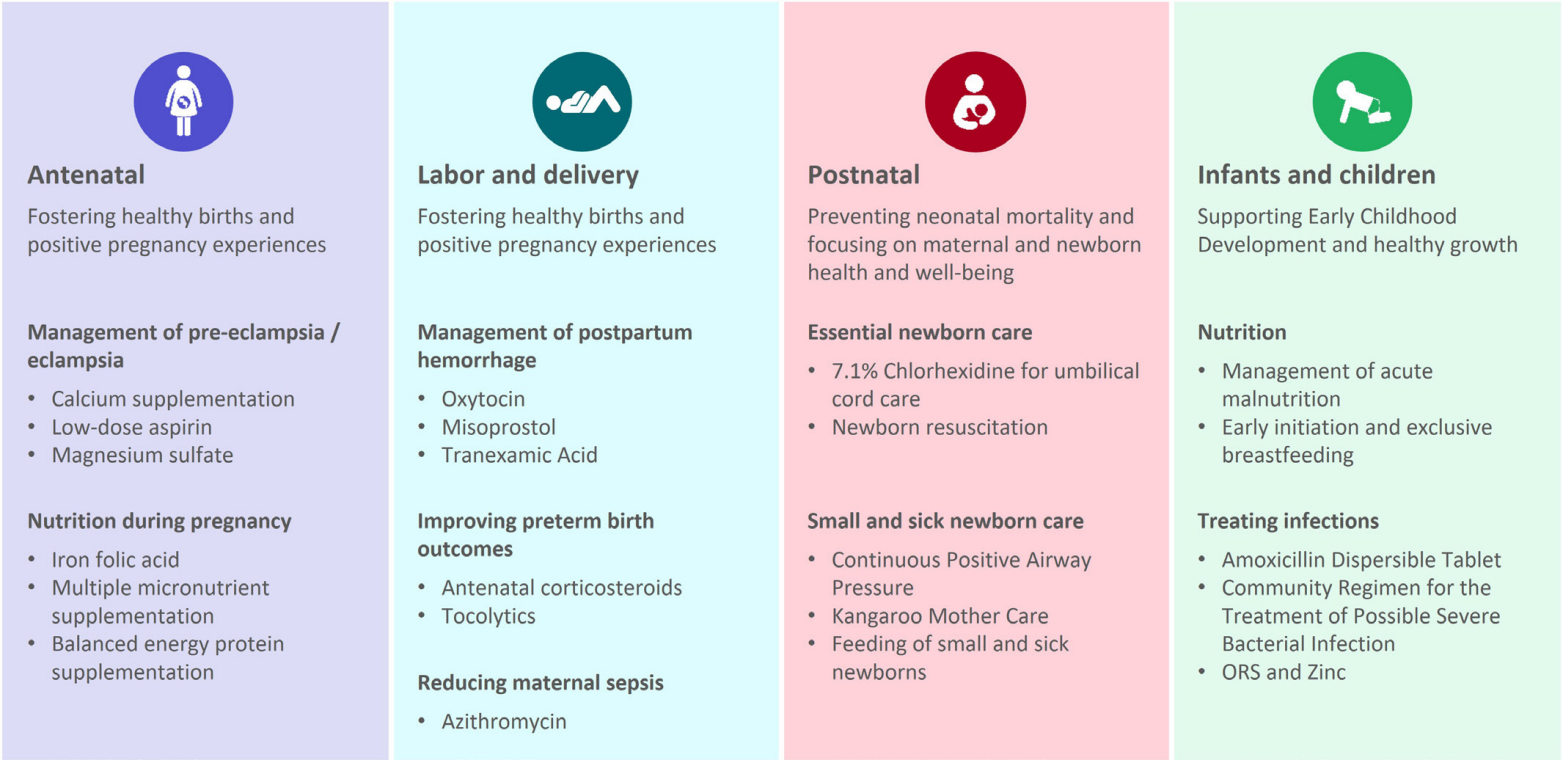
MNCHN interventions, by stage of life course. MNCHN, maternal, newborn, child health and nutrition; ORS, oral rehydration salts.

Intervention selection was determined by the donor, based on their strategic interests and included 22 maternal, newborn, child health and nutrition interventions (figure 2) across the life course (and is not inclusive of all high-impact interventions).

## Visualising the framework data to contextualise country progress and inform action

We sought to create a searchable dynamic database as a global good to help inform global and national efforts, accompanied by analysis and synthesis to bring the data to life. Once we collected all the publicly available data for the intervention, we used qualitative findings from the key informant interviews to triangulate and pinpoint the stage of scale for each intervention, by country. We identified which indicators and data exist to measure the milestones and created interactive dashboards to summarise progress across interventions and countries. In total, our database includes over 83 000 data points that align to this framework and have been sourced from key informant interviews, document review, and global or country surveys (data points are uniquely defined by country, framework milestone number, indicator name, indicator value, data source, year). Survey indicators aligning to this framework were pulled from a variety of sources including: DHS indicators (n=36 607), SARA indicators (n=156), SPA indicators (n=70), WHO SRMNCAH policy survey indicators (n=6199), ENAP (n=1060), USAID/MOMENTUM indicators (n=598) and Harmonised Health Facility Assessments (n=169) among other national surveys. The dashboard is publicly accessible via PATH’s website.^11^

**Figure 3:**
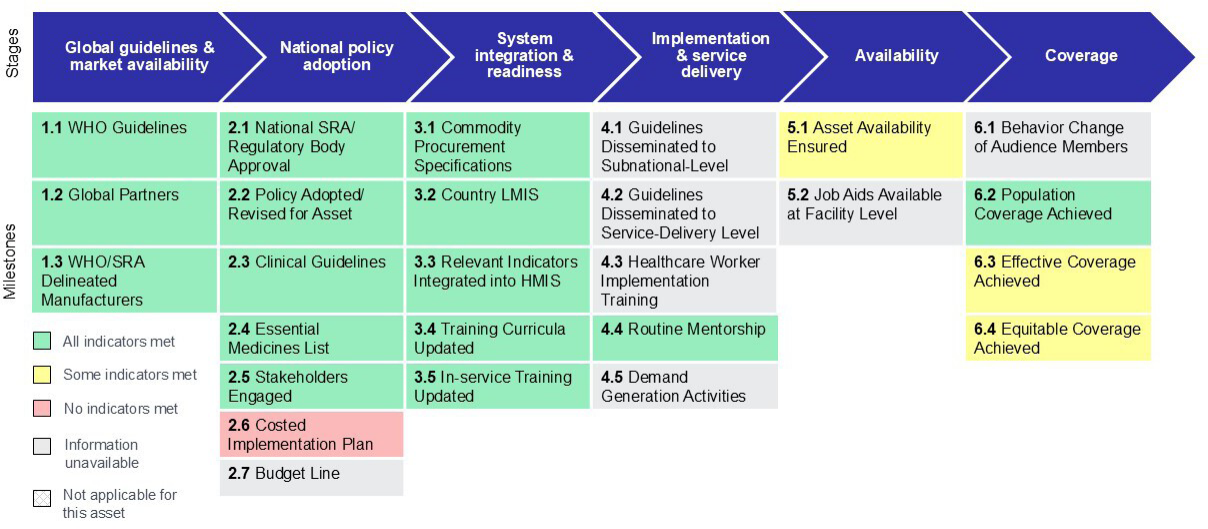
Kenya’s progress on achieving the milestones towards coverage of IFA. HMIS, health management information system; IFA, iron folic acid; LMIS, logistics management information system; WHO, World Health Organization; SRA, Stringent Regulatory Authority.

From a practice perspective, the visualisation showing country progress on achieving milestones towards effective coverage (figure 3) may be the most useful, as it assists country planners in deciphering the most immediate next steps needed to advance the scale of a particular intervention within a specific country context, as well as rapidly identifying data gaps. For example, figure 3 pulls the most recent data points from source documents, key informant interviews, and multiple surveys into one location and visualises the existing data to show progress towards the scale of iron folic acid (IFA) in Kenya. When accessing the web-baseddashboardtool, hovering over each milestone in the framework will reveal the underlying data sources, indicators and values.^11^ With respect to national policy adoption, IFA has regulatory market authorisation, a national policy has been adopted for IFA, and clinical guidelines exist (milestones 2.1, 2.2, 2.3). IFA is listed within the 2023 national essential medicines list (category: anti-anemics) enabling government procurement (milestone 2.4) and IFA is covered by the national technical working group on reproductive and maternal health (milestone 2.5). A costed implementation plan specific to IFA was not clearly earmarked (milestones 2.6, 2.7). Within the systems domain, commodity procurement specifications exist (milestone 3.1) and updated national guidelines and clinical protocols integrating IFA support the in-service and preservice training curricula (milestones 3.4, 3.5); in addition, relevant indicators have been integrated into the logistics management information system (LMIS) and health management information system (HMIS) (ie, ANC clients given IFA) (milestones 3.2, 3.3). Regarding implementation, information could not be identified to track guideline dissemination to the subnational or service-delivery levels (milestones 4.1, 4.2), or the number of health care worker trainings or number trained on IFA services (milestone 4.3). Availability indicators exist in the Kenyan Harmonized Health Facility Assessment (2018) survey tracking the per cent of facilities offering iron (79%) and/or folic acid (77%) supplementation within ANC (milestone 5.1).^23^ For coverage, indicators exist to track population coverage (90.2% of pregnant women took any iron-containing supplements), effective coverage (by user adherence to guidelines, 54.6% of pregnant women took 90+ days iron-containing supplements) and equitable coverage (by wealth quintile, education, urban/rural residence) (milestones 6.2, 6.3, 6.4).^24^

The interactive dashboards are designed to assist with cross-country comparisons as well as within-country cross-intervention comparisons, and to inform the development of key actions and next steps for advancing scale-up, including gaining consensus on the way forward. For example, in Burkina Faso, following a dissemination event to review the intervention data and insights, the government took immediate action to draft a roadmap outlining key actions and responsible entities to address barriers to scale for selected interventions. Similarly, in Kenya, during the dissemination event, the Ministry of Health shared the importance of integrating the key insights from these dashboards and accompanying analyses into national documents to ensure broad use of the tool and findings by policy-makers and partners—specifically, integrating the data into the Kenya Health Sector Strategic Plan and engaging the Council of Governors in disseminating the insights to the county level. An accompanying how-to resource supports users, including advocacy organisations, in reviewing, packaging and presenting data from PATH’s MNCHN Asset Tracker to inform local advocacy efforts.^25^

## Data consistency and quality

Data compiled underlying the framework represents a range of accessibility, availability and quality measures, influenced by the country and type of data source. Challenges with accessing information limit aspects of this data tracker. For example, obtaining routine country data from HMIS and LMIS generally requires agreement from country governments to ensure data can be accessed, pulled from the system, and used/presented, even for aggregate-level data. Data pulls from repeated surveys at household/facility or country level can often require permission, approvals or data sharing agreements. Additionally, survey years and frequency vary considerably between countries; therefore, data across countries are not comparable. The quality of data used in the accompanying dashboards is also uneven as the indicators vary in consistency, comparability and accessibility. For example, data comparability across countries is high for global databases such as the WHO SRMNCAH policy survey database since it is derived from a standard global survey administered online to member states and uses consistent indicator names to ensure indicator comparability across countries.^26^ On the other hand, data comparability for routine country data (HMIS) is moderate since indicator definitions are not consistent for a wide-ranging set of indicators, and overall data quality can be limited. These challenges with facility-derived data are consistent with other reports of the need to improve routine data quality used in estimated coverage levels.^27^ Finally, maintaining the dashboard for functionality, as well as updating to ensure data validity can be resource-intensive.

Recent literature points to nuance and evolution in the ‘effective coverage’ concept,^28^ and given the measurement complexity and resource requirements, researchers have begun exploring the utility of secondary data sources, such as the DHSs, in assessing effective coverage.^29 30^ In terms of measuring effective coverage, we found large data gaps for each intervention within secondary data sources. Our experience is consistent with a recent rapid systematic review of 33 studies that measured effective coverage and cascades for childbirth, newborn and child health in low-income and middle-income countries^31^ and found that effective coverage measures (both outcome adjusted and quality adjusted) can be challenging to construct due to data gaps and non-standardisation of indicators across geographies. However, despite these disparate data sources and limitations, the dashboards pair a pragmatic framework with aggregated data visualisations that illustrate the journey towards scale for specific evidence-based interventions. Through visually identifying where along the pathway to scale an intervention has progressed, the framework can serve to spark discussions among key stakeholders around where to target implementation strategies to advance the intervention further towards effective coverage.

## Conclusion: the road ahead for monitoring scale

Increasing availability and coverage of essential MNCHN commodities is core to advancing universal health coverage, but progress in improving health service access and capacity has been minimal since 2015, in part due to financing gaps for MNCHN relative to other health areas such as HIV and malaria.^21 32^ A renewed commitment in 2022–2023 by countries and technical partners to accelerate progress on scaling a prioritised set of maternal and newborn health commodities (building from the 2012 UN Commission on Life-saving Commodities for Women and Children)^33^ has highlighted the need for facilitating strong implementation strategies and in supporting countries to monitor scale-up progress.^34^ Our proposed framework can be used to support this effort by addressing the need for an easy-to-follow implementation pathway and a set of common policy and implementation indicators to monitor scale-up towards effective coverage that uses existing secondary data sources where available.

This framework can also be extended to other health domains and be used to customise (eg, at the subnational level, if suitable data are available) a monitoring approach tailored to where more focus is needed, for example, either through pinpointing where progress has stalled, and/or where data gaps indicate more resources are required to measure key milestones along the pathway to scale—both of which can help in supporting intervention-specific roadmaps, costed plans and further action planning. Key uses of this framework in any domain could include supporting policy dialogue and planning for introduction and scale of high-impact interventions as well as monitoring and evaluation of their impact.

Our framework was designed to assess individual interventions, but as more countries adopt a life-course approach consistent with primary healthcare, framework adaptations will be needed to capture bundled intervention packages for prepregnancy, pregnancy, birth, postnatal, neonatal, child and adolescence.^35^ The framework could also benefit from additional sensitivity analyses to hone the milestones that are most meaningful for tracking scale, and future evolutions could consider the incorporation of respectful care (eg, person-centred maternity care) and additional dimensions of equity,^36^ as well as detailed theories of change to link milestones to measures of effective coverage.

Call-to-action: Achieving effective, equitable coverage to reduce maternal and child mortality will require increased commitment and investment to collect transparent data on commodity availability and uptake, and critically, to use that data for implementation monitoring, action planning and to drive progress towards scaling lifesaving interventions.

## Supplementary material

10.1136/bmjgh-2024-016494 online supplemental file 1

## Data Availability

Data are available in a public, open access repository.
