# Supplementary material for: Developing a framework for monitoring the stages towards achieving effective coverage and equity for maternal, newborn, child health and nutrition interventions
Source: BMJ Glob Health. 2025 Apr 9;10(4):e016494. doi: 10.1136/bmjgh-2024-016494 (PMC11987099; doi:10.1136/bmjgh-2024-016494)
Supplement: online supplemental file 1 [file bmjgh-10-4-s001.pdf]

Supplementary Table A. The complete list of 38 frameworks included in our literature review.

**Theoretical framework/model**	**Core concepts**
ExpandNet Framework (WHO)	The framework provides a way of systematically thinking about scaling up and consists of five elements inside the oval with the scaling-up strategy as the centrepiece and five strategic choice areas (the boxes outside the oval). The framework is guided by four key principles which are: systems thinking; a focus on sustainability; the need to determine scalability; and respect for gender, equity and human rights principles.
PARIHS Model (Promoting Action on Research Implementation in Health Services)	The PARIHS model emerged from the observation that successful implementation in health care might be premised on three key determinants: characteristics of the evidence, context and facilitation.
AAAQ model	Focuses on four components for scaling up an intervention: Accessibility, Availability, Affordability, Quality
CFIR (Consolidated Framework for Implementation Research)	The CFIR is composed of five major domains: intervention characteristics, outer setting, inner setting, characteristics of the individuals involved, and the process of implementation. Eight constructs were identified related to the intervention (e.g., evidence strength and quality), four constructs were identified related to outer setting (e.g., patient needs and resources), 12 constructs were identified related to inner setting (e.g., culture, leadership engagement), five constructs were identified related to individual characteristics, and eight constructs were identified related to process (e.g., plan, evaluate, and reflect).
WHO's health system building blocks framework	WHO framework that describes health systems in terms of six core components or "building blocks": (i) service delivery, (ii) health workforce, (iii) health information systems, (iv) access to essential medicines, (v) financing, and (vi) leadership/governance
Ely's Conditions of Change Model	Describes eight conditions that facilitate the adoption, implementation, and institutionalization of educational technology innovations and suggests applications to the transfer of portable software
MNCH continuum of care model	Model which promotes integrated service delivery throughout the life cycle
ADEPT model	Aims to explain and influence policy development and policy impact implementation with four determinants: goals, obligations, resources and opportunities.
Tanahashi Model for Achieving Effective Coverage	Uses five important stages that successively lead to a desired health intervention and to define measurements of coverage appropriate to these stages: availability coverage, accessibility coverage, acceptability coverage, contact coverage, effective coverage.
IHI Framework for Going to Full Scale	The framework describes three core components: a sequence of activities that are required to get a program of work to full scale, the mechanisms that are

	required to facilitate the adoption of interventions, and the underlying factors and support systems required for successful scale-up. The four steps in the sequence include (1) Set-up, which prepares the ground for introduction and testing of the intervention that will be taken to full scale; (2) Develop the Scalable Unit, which is an early testing phase; (3) Test of Scale-up, which then tests the intervention in a variety of settings that are likely to represent different contexts that will be encountered at full scale; and (4) Go to Full Scale, which unfolds rapidly to enable a larger number of sites or divisions to adopt and/or replicate the intervention.
Ecological Framework	The ecological framework is based on evidence that no single factor can explain why some people or groups are at higher risk of interpersonal violence, while others are more protected from it. This framework views interpersonal violence as the outcome of interaction among many factors at four levels—the individual, the relationship, the community, and the societal.
Diffusion of Innovations model	In this concept, innovations were defined as ideas or practices perceived as new by practitioners (in this case, farmers). Diffusion was seen as the spread of ideas among individuals, largely by imitation. Interventions aimed at spreading innovation harnessed the interpersonal influence of opinion leaders and change agents, and research mapped the social networks and adoption decisions of targeted individuals. Furthermore, the Theory of Diffusion highlights the importance of intermediary actors (opinion leaders, change agents and gatekeepers) for successful adoption and implementation
Understanding-User-Context Framework	The framework contains five domains that should be taken into consideration when establishing interactions with users: the user group; the issue; the research; the research-user relationship; the dissemination strategies. Each domain includes a series of questions. The purpose of these questions is to provide a way to organize what the researcher already knows about the user group and knowledge translation, identify what is still unknown, and flag what is important to know.
Theoretical domains framework	It was constructed on the basis of a synthesis of 128 constructs related to behaviour change found in 33 behaviour change theories, including many social cognitive theories. The constructs are sorted into 14 theoretical domains (e.g. knowledge, skills, intentions, goals, social influences and beliefs about capabilities). Theoretical Domains Framework does not specify the causal mechanisms found in the original theories, thus sharing many characteristics with determinant frameworks.
Active Implementation Frameworks (AIFs)	Set of frameworks to use when attempting to put into practice any innovation of known dimensions
Gurses et al model	Draws upon many theories/frameworks to provide a comprehensive and systematic guide to identify barriers to guideline compliance (namely in the clinical setting)
Implementation Climate Theory	Our model suggests that implementation effectiveness-the consistency and quality of targeted organizational members' use of an innovation-is a function of (a) the strength of an organization's climate for the implementation of that innovation and (b) the fit of that innovation to targeted users' values. The model specifies a range of implementation outcomes (including resistance, avoidance, compliance, and commitment); highlights the equifinality of an

	organization's climate for implementation; describes within- and between-organizational differences in innovation-values fit; and suggests new topics and strategies for implementation research.
Behavior Change Wheel	At the centre of this framework is a 'behaviour system' involving three essential conditions: capability, opportunity, and motivation (what we term the 'COM-B system'). This forms the hub of a 'behaviour change wheel' (BCW) around which are positioned the nine intervention functions aimed at addressing deficits in one or more of these conditions; around this are placed seven categories of policy that could enable those interventions to occur.
Bloom's Taxonomy of Learning	consisted of six major categories: Knowledge, Comprehension, Application, Analysis, Synthesis, and Evaluation. The categories after Knowledge were presented as "skills and abilities," with the understanding that knowledge was the necessary precondition for putting these skills and abilities into practice.
Organizational readiness for change theory	The theory described in this article treats organizational readiness as a shared psychological state in which organizational members feel committed to implementing an organizational change and confident in their collective abilities to do so. This way of thinking about organizational readiness is best suited for examining organizational changes where collective behavior change is necessary in order to effectively implement the change and, in some instances, for the change to produce anticipated benefits.
Conceptual framework for vaccine coverage	Identifies three principal determinants of vaccine coverage: Intent to Vaccinate, Health Facility Readiness and Community Access
Conceptual framework of access to healthcare	This framework conceptualizes five dimensions of accessibility: 1) Approachability; 2) Acceptability; 3) Availability and accommodation; 4) Affordability; 5) Appropriateness. In this framework, five corresponding abilities of populations interact with the dimensions of accessibility to generate access. Five corollary dimensions of abilities include: 1) Ability to perceive; 2) Ability to seek; 3) Ability to reach; 4) Ability to pay; and 5) Ability to engage
Conceptual model of health systems governance	A people-centred health systems governance conceptual model based on applying four effective governing practices: cultivating accountability, engaging with stakeholders, setting a shared strategic direction, and stewarding resources responsibly. This utilizes a participatory approach where health system leaders identify and act on opportunities for making themselves and their health systems more accountable and responsive to the needs of the communities they serve.
Primary Health Care Performance Initiative (PHCPI) Framework	More than a package of services, quality PHC has 4 core functions: comprehensiveness of promotive, preventive, curative, and palliative care services; continuity across the life cycle; coordination across service providers and levels of the health care system; and a point of first contact access for the majority of patients' health needs. It has 5 domains: System, Inputs, Service Delivery, Outputs, and Outcomes, and each has 5 or more subdomains and sub-subdomains
PRISM model	PRISM—the Practical, Robust Implementation and Sustainability Model—evaluates how the health care program or intervention interacts with the

	recipients to influence program adoption, implementation, maintenance, reach, and effectiveness
Sabatier and Mazmanian Framework	Framework which describes processes from development of a health policy to implementation and performance.
Organizational Change Framework	Organisational Transformation Model comprises six components that are critical for a successful transformation project. Each component in turn comprises three elements. Experience and research have taught us that for every element that is missing, the probability of project failure increases.
MCSP Conceptual Framework	Program conceptual framework which aims to provide a process for accelerating innovations to scale. It defines five stages (inputs, processes, outputs, outcomes, impact at scale) to be accomplished across the RMNCAHN continuum of care.
Absorptive Capacity Framework	Framework which defines the amount and form of international aid and attention that recipient institutions and societies can receive without suffering significant social, economic, or po liti cal disruptions.
IHI Framework for Spread	Identifies six areas for achieving spread: leadership, establishment of better ideas, strengthening the social system, initial spread strategy, use of data to guide spread, refinement.
Action Learning Framework	Framework for process improvement, which marries analysis and action, reflection and doing, organizational change and bottom-line performance.
Social ecological model	The social ecological model (SEM) emphasizes that behavior affects and is shaped by one's environment. It highlights that a behavior is molded by the following levels of influence: individual; interpersonal (family, friends, peers); community (community leaders, organizations, healthcare services); and societal (policy/legislation, religion, social and gender norms). Factors that indirectly affect the behavior make up the enabling environment.
Theory of Reasoned Action/Planned Behavior	The Theory of Reasoned Action emphasizes that behavioral intentions lead to the behavior, which are based on two things: attitudes toward the behavior and subjective norms (beliefs about whether people around you perform the behavior and how that affects whether you will practice it). The Theory of Reasoned Action was later expanded to include perceived behavioral control (belief that one can perform the behavior) as an influencer of behavioral intention, forming the Theory of Planned Behavior.
Stages of Change (Transtheoretical Model)	The Theory of Reasoned Action emphasizes that behavioral intentions lead to the behavior, which are based on two things: attitudes toward the behavior and subjective norms (beliefs about whether people around you perform the behavior and how that affects whether you will practice it). The Theory of Reasoned Action was later expanded to include perceived behavioral control (belief that one can perform the behavior) as an influencer of behavioral intention, forming the Theory of Planned Behavior.
Social Learning Theory	The Social Learning Theory posits that people learn how to behave by 1) observing others' behavior, 2) observing the apparent consequences of those behaviors, 3) assessing those consequences in their own lives, 4) trying out the behavior themselves.
Social Cognitive Theory	The Social Cognitive Theory evolved out of the Social Learning Theory, utilizing the principles of observational learning and outcome expectancies. It broadened the theoretical framework of Social Learning Theory by

	emphasizing that observation of other’s actions works to increase others’ self-efficacy, thereby improving their likelihood of performing a behavior. Collectively, Social Cognitive Theory notes that one’s environment provides models for behavior, and the reinforcement a person receives from the people around them and their environment can increase or decrease the likelihood of them performing a behavior.
Health systems evaluation framework for Tanzania countdown to 2015 case study	Health systems evaluation framework to assess coverage and equity of interventions along the continuum of care, health systems, policies and investments, while also considering contextual change (eg, economic and educational).
IMCI impact model	Systematic model of scaling up the IMCI strategy
